# Factors influencing disability in patients with chronic low back pain attending a tertiary hospital in sub-Saharan Africa

**DOI:** 10.1186/s12891-019-2403-9

**Published:** 2019-01-15

**Authors:** Marie Doualla, Jeannine Aminde, Leopold Ndemnge Aminde, Fernando Kemta Lekpa, Felix Mangan Kwedi, Emmanuel Vubo Yenshu, Alain Mefire Chichom

**Affiliations:** 1 Faculty of Health Sciences, University of Buea, Buea, Cameroon; 2 Faculty of Medicine and Pharmaceutical Sciences, University of Douala, Douala, Cameroon; 3 Douala General Hospital, Douala, Cameroon; 4 Faculty of Social & Management Sciences, University of Buea, Douala, Cameroon; 5 Faculty of Medicine, School of Public Health, The University of Queensland, Brisbane, Australia

**Keywords:** Chronic low back pain, Disability, Africa

## Abstract

**Background:**

Very little is known about the burden of chronic low back pain in Africa. This study aimed at assessing disability and associated factors in chronic low back patients in Cameroon.

**Methods:**

We carried a hospital-based cross-sectional study including patients suffering from low back pain (LBP) of at least 12 weeks’ duration. Disability was assessed using the Roland Morris Disability Questionnaire (RMDQ). RMDQ > 4 described persons with dysfunctional levels of disability.

Multivariable linear regression was used to investigate factors associated with higher RMDQ scores hence greater disability. Variables investigated included; gender, age, marital status, employment status and type, smoking history, alcohol consumption, income, pain intensity, LBP duration, psychological wellbeing, sleep satisfaction, leg pain, numbness/paresthesia, bowel/bladder dysfunction symptoms (BBDS), body mass index (BMI), and days of work absence.

**Results:**

A sample of 136 adults (64% female) with a mean age of 50.6 ± 12.2 years participated in the study. Median duration of LBP was 33 (25th – 75th percentile: 12–81) months. Mean RMDQ score was 12.8 ± 6. In multivariable linear regression, pain intensity (β = 0.07, *p* = 0.002), longer days of work absence (β = 0.15, *p* = 0.003) and BBDS (β =2.33, *p* = 0.029) were associated with greater disability. Factors such as consumption of alcohol (β = − 3.55, *p* = 0.005) and higher psychological wellbeing scores (β = − 0.10, *p* = 0.004) significantly contributed to less disability (lower RMDQ scores). Dysfunctional levels of disability were present in 88.1% of patients.

**Conclusion:**

CLBP is associated with significant disability and this relationship is driven by several factors. Multidisciplinary management strategies especially those targeted to improve pain control, manage BBDS and improve psychological wellbeing could reduce disability and improve quality of life.

## Background

LBP is described as “pain, muscle tension, or stiffness localized below the costal margin and above the inferior gluteal folds, with or without leg pain (sciatica), and is defined as chronic when it persists for 12 weeks or more” [1]. It is estimated that, at any point in time, about 11.9% of the world’s population is suffering from LBP [2] . The prevalence of chronic LBP worldwide is estimated at 19.6% in those aged between 20 and 59 years [3].

In Africa, LBP is increasingly recognised as a major health problem. A systematic review of epidemiological studies across Africa reported a pooled adult prevalence of 32% with an average lifetime prevalence 62% [4]. This is higher than the 28.8% reported among adult Americans in 2013 [5]. In Cameroon, the prevalence of CLBP was 19.1% among patients presenting for rheumatology consultations during 2004 to 2013 at the Douala General Hospital [6].

Interestingly, though a frequent cause of clinic visits, the specific aetiology of the pain in LBP is not often identified; in which case it is referred to as “non-specific LBP”. Therefore, the first aim of the clinical evaluation is usually to situate the patient in one of three categories; non-specific low back pain, back pain with radiculopathy or stenosis, and back pain associated with another specific spinal cause [7]. It is equally considered important to identify in the patient any risk factors for progressing to chronic disability [7], LBP being a well-recognised disabling condition [8]. Disability refers to a restriction or lack of ability to perform an activity in the manner or within the range considered normal for a human being [9]. Disability is a core issue in LBP, affecting physical performance and consequently work productivity [10].

According to the Global Burden of Disease (GBD) 2015 study, LBP is the leading cause of disability worldwide, accounting for 815 Years Lived with Disability (YLD) per 100,000 populations. This value represents a 17.2% increase since 2005 [8]. This study reported that lower back and neck pain grouped, constituted the leading cause of disability in all high-income countries, and in almost all Latin American, Asian, and Middle Eastern countries.

In Sub-Saharan Africa (SSA), a Ugandan hospital based cross-sectional described significant disability in LBP patients with 87% of participants reporting up to 14 days of work loss due to LBP [11]. In nine of the countries in this region, LBP and neck pain are recognized as the leading causes of YLD (2). Cameroon is one of these nine and LBP is also the first cause of rheumatologic consultation [6].

A number of methods assessing disability in LBP have been described in the literature, broadly classified as self-report or performance-based measures. Performance-based tests like the Isernhagen Work Systems Functional Capacity Evaluation (IWS-FCE) are thought to provide an objective representation of a patient’s functional capacity [12, 13], and hence are frequently used in rehabilitation medicine. On the other hand, self-report tools (mostly questionnaires) are more practical, inexpensive and are widely used in CLBP research. The RMDQ is a self-report back pain specific disability tool that measures limitation in activities of daily living. It has been found sensitive and reliable [14], and has been validated in patients with low back pain across different settings including West African communities [15]. It is the second most widely used tool of its kind after the Oswestry Disability Questionnaire. In comparison to the Oswestry tool, the RMDQ has been found simpler to use, readily understood with similar psychometric properties [16–18].

Studies in Brazil (17, 18) and in Egypt [21] among patients with CLBP have reported mean RMDQ scores generally above 14 (out of the total score of 24). However, in a study in Netherland a slightly lower score (12.6) was reported in patients in a pain management centre [22].

Core muscle dysfunction is believed to be a major trigger for low back pain [23, 24]. These muscles are responsible for maintaining spine stability and counteracting external forces. Weakness results in instability and strain on the vertebral column and intervertebral discs [23]. Core muscle dysfunction has equally been associated with more severe pain and greater disability in LBP patients [24, 25].

Like core muscle dysfunction, impaired psychological wellbeing and impaired sleep quality are recognized risk factors of LBP (and possibly also consequences of the pain), while equally prolonging disability in these patients [26–28]. Fear of movement from fear of the pain or re-injury results in muscle disuse and structural changes, hence another cause of prolonged disability in LBP [27, 29, 30]. More so, leg pain, back tenderness, lack of exercise and advancing age have equally been associated with greater disability in LBP [30, 31].

In Cameroon, there are no published studies exploring disability among CLBP patients. This represents an immense knowledge gap that this study sought to fill. The purpose of this study was thus to assess the level of and factors associated with disability among patients with CLBP. Our goal was to provide a better understanding of the factors that contribute to disability in CLBP patients in Cameroon in order to inform management strategies.

## Methods

### Study design and setting

This was a cross-sectional study conducted during January to March 2017 at the Rheumatology unit of the Douala General Hospital (DGH) in Cameroon. Three rheumatologists (who actually made or confirmed the diagnoses for patients in the study) are responsible for this unit. The unit runs outpatient consultations from Monday to Friday. Each rheumatologist has two consultation days, with an average of 1 or 2 rheumatologists consulting daily. Each rheumatologist consults approximately 15–30 patients a day, 20–40% of which are low back pain patients presenting either de novo or for follow-up visits. DGH is a tertiary referral and teaching hospital receiving patients from all ten regions in Cameroon and surrounding African countries. Douala, the economic capital is the most populated city of Cameroon with an estimated population of 2,768,400 in 2015 [32].

### Sampling and study participants

We consecutively included adult patients aged 18–70 years with LBP of at least 12 weeks’ duration who presented de novo or for follow up visits during the study period. LBP was defined as: sensations of pain, muscle tension, or stiffness, localized below the costal margin and above the inferior gluteal folds. The area involved identified on a human diagram. Patients with pregnancy, and suspicion of cauda equina syndrome, recent trauma or LBP surgical emergencies, and patients unable to comprehend questions were excluded.

### Study procedures and data collection

Eligible and consenting participants were invited to complete an interviewer-administered semi-structured questionnaire. This was available in English and French (the two official languages in Cameroon) to serve participants expressing themselves in either language. Data on general characteristics obtained included; gender, age, marital status (single, married or widowed), employment status (employed, housewife, student, unemployed or retired), employment type (physical labour, non-physical labour), smoking history (current smoker, former smoker and non-smoker), alcohol consumption (consumer or non-consumer), quantity of alcohol consumed (expressed in units per week). An “alcohol consumer” was considered to be a study participant who admitted to consuming at least one alcoholic drink in the month preceding the interview. Other data included level of education (no education, primary, secondary and tertiary education) and average monthly income (< 50,000 FCFA, 50000–100000FCFA, 100,000–300,000 FCFA, > 300,000 FCFA [1US$ = 530FCFA]).

### Assessment of disability

Disability was assessed using the Roland Morris Disability Questionnaire (RMDQ), a 24-item LBP-specific tool that assesses impairment in activities of daily living. The RMDQ was chosen for its low administrative burden, easy comprehensibility, proven high responsiveness and sensitivity in LBP, and the availability of validated versions in English and French [33]. It is scored from zero to 24, with higher scores implying greater disability. In this study, we further categorized participants as dysfunctional (RMDQ score > 4) or functional (RMDQ score ≤ 4). A score of four is a proposed cut-off to classify LBP patients as functional or dysfunctional [34], and is supported by the findings of other authors [35].

### Other clinical characteristics

*Pain intensity* was measured with a 100 mm visual analogue scale (VAS). Participants were required to report the *total duration of their CLBP* by answering the question; “For how long have you had an ongoing low back pain problem?”, adapted from the recommendations of the CLBP Research Task Force of the American National Institute of Health Pain Consortium [36]. They equally reported the *duration of the current pain symptoms/episode* by answering the question “How long has it been since you went for a whole month without low back pain?”, based on the definition of a LBP episode by Vet et al. [37]. This was done in an effort to clearly characterize the duration of pain, taking into account the challenges that exist due to the often intermittent, recurring nature of LBP, and considering the ambiguity in existing LBP terminology. Participants were asked to report the presence or absence of certain symptoms; *leg pain, numbness/paresthesia* in lower limbs (any of; tingling, burning, electric-currents, numbness, pins and needles); *BBDS* or *bladder/bowel dysfunction symptoms* (any of; uncontrollable urges to urinate or stool, urine or stool leakages; straining unduly when stooling or initiating urine).

*Psychological wellbeing* of participants was assessed using the psychological domain score of the World Health Organization Quality of Life brief tool (WHOQOL-BREF). This is a generic tool that assesses quality of life with six measures. The psychological domain score is computed by summing scores of seven specific items covering bodily image and appearance, negative feelings like anxiety and depression, positive feelings, self-esteem, spirituality/religion/personal beliefs, and thinking, learning, memory and concentrations. The summed score is transformed to a scale of 0–100 using the steps described in the WHOQOL manual [38], with higher scores indicating greater wellbeing.

S*leep satisfaction* was assessed by asking patients to rate their satisfaction with their sleep in the past month on a scale of 1 to 5 (1 representing ‘very dissatisfied’ and 5 representing ‘very satisfied’). This was expressed as a percentage score.

Patients’ *weight* and *height* were also measured. This was done with participants wearing light clothing and without shoes; their weight was measured using Seca® scales while height was measured with the adult Leicester® stadiometer. The stadiometers were placed against the wall, while participants stood upright without their shoes and their heels and occiput on the stadiometer. For height, measures were to the nearest 0.5 cm while for weight we considered one decimal place. Their hospital medical records were reviewed to collect information on radiologic findings and treatment.

*Work absence due to LBP* was denoted “days of work loss” and defined as the number of days of restricted routine activity (inability to carry out your regular activities) or absence at workplace because of CLBP occurring within the 30 days preceding the interview.

### Ethical considerations

This study received Ethical Approval from the Faculty of Health Sciences Institutional Review Board (2017/003/UB/SG/IRB/FHS). The components and purpose of the study were explained to all potential study participants and only those who freely gave written consent were included. Patient confidentiality was maintained and the study adhered to the World Medical Association’s Declaration of Helsinki.

### Data management and statistical analysis

Data were analyzed using the Statistical Package for Social Sciences (SPSS Inc., Chicago, Illinois, USA) version 20. Results are summarized as counts and percentages for categorical variables and as means and standard deviation (SD) or median with 25th and 75th percentiles where appropriate for continuous variables. Standard assumptions of parametric tests (e.g. linearity, normality of residuals, homoscedasticity) were tested; as such, we performed no data transformations. The RMDQ score was analyzed as a continuous outcome variable. We adopted a threshold score of ≥4 to report prevalence of dysfunctional CLBP, based on a previous study conducted among adult LBP patients [34]. Bivariate analysis was performed to investigate significant associations with RMDQ scores/disability. Pearson’s correlation was used for continuous variables and for categorical variables, the independent samples t-test was used to test the group differences in mean RMDQ scores and in case of more than two groups, analysis of variance (ANOVA) was used. Variables that were significant, or trending towards (*p* < 0.1) in bivariate analysis, were then fitted in a multivariable linear regression model to determine factors independently associated with disability. Prior to fitting the multivariable model, we checked for evidence of multicollinearity in the independent variables via a correlation matrix and then ran collinearity diagnostics to assess their tolerance and variance inflation factors (VIF). All VIFs were less than 2, suggesting absence of any multicollinearity. Statistical significance was set at *p* < 0.05.

## Results

### General characteristics

**Fig. 1:**
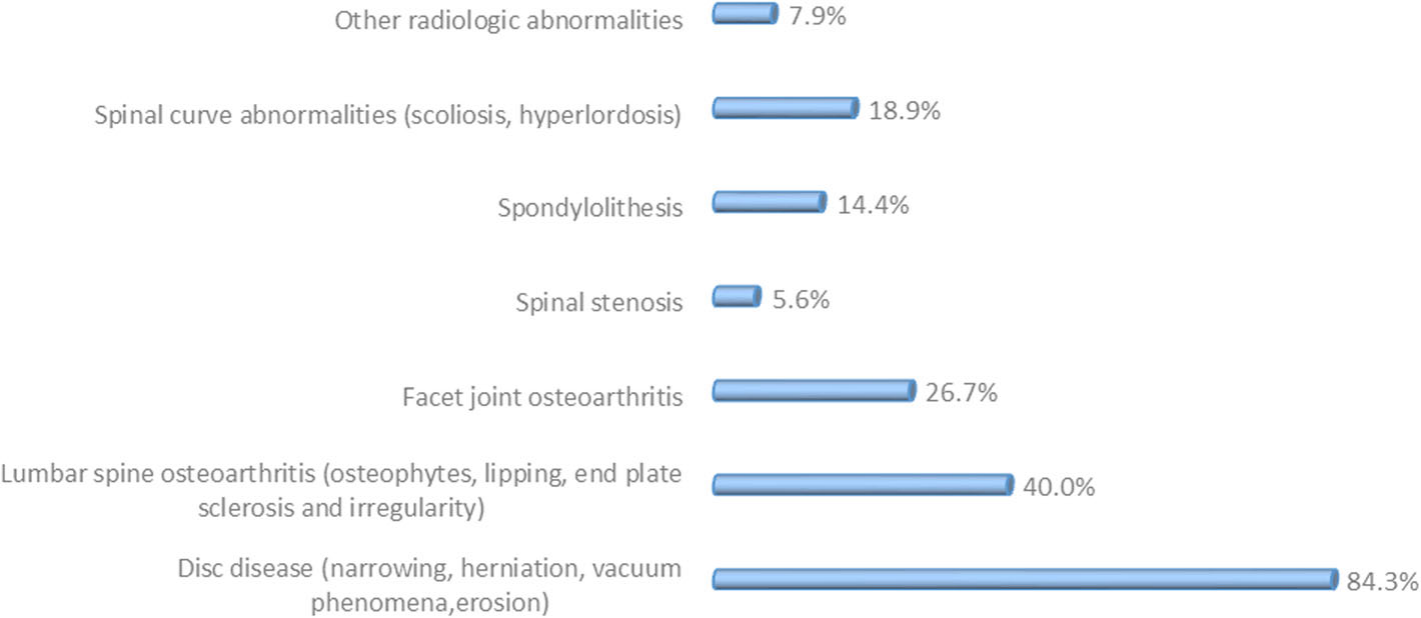
Specific diagnoses of patients with CLBP. Legend: Bar chart showing the different aetiological diagnoses of patients with CLBP. The percentages next to the bars represent the proportion of each diagnosis in the cohort of patients.

A sample of 136 CLBP (64% females) was included. Forty-one percent of participants had non-specific CLBP, 56% CLBP with radiculopathy/stenosis, and 3% had CLBP from a specific spinal cause. The principal specific aetiologies encountered in participants are described in Fig. 1. The mean age was 58.7 years. Seventy-three percent were married and about two-thirds (65%) had secondary education level or less. Most (71%) were employed, and work type generally involved no physical labour (69%). Forty-five percent of participants earned > 100,000 FCFA (> 190$), Table 1.

**Table 1:** General characteristics of the study population

Variable	Category	n	%
*Gender*	Male	48	35.6
	Female	87	64.4
*Marital Status*	Married	97	73
	Single	24	18.0
	Widowed	12	9.0
*Educational level*	No formal	2	1.5
	Primary	32	23.5
	Secondary	54	39.7
	Tertiary	48	35.3
*Employment status*	Unemployed	14	10.4
	Employed	95	71.1
	Student	3	2.2
	Housewife	13	9.6
	Retired	9	6.7
*Employment type*	Physical labour	26	27.1
	Non-physical	66	68.8
	Combination	4	4.2
*Income level (FCFA)*	< 50,000	56	41.5
	50,000–100,000	18	13.3
	100,000 – 300,000	28	20.7
	> 300,000	33	24.4
*Alcohol*	Non-consumer	27	19.9
	Consumer	109	80.1
*Smoking*	Non-smoker	112	82.4
	Former	21	15.4
	Current	3	2.2
*Sensory neuropathy*	Absent	67	49.3
	Present	69	50.7
*BBDS*	Absent	90	66.2
	Present	46	33.8
*Leg pain*	Absent	59	43.4
	Present	77	56.6
*Treatment*	No	22	16.7
	Yes	110	83.3

*BBDS* = Bowel/bladder dysfunction symptoms, *FCFA* = Central African Franc

### Pain, disability and work loss

The median (IQR) duration of CLBP was 33.0 (69) months. The median duration of current pain symptoms was 12 months and median pain intensity was 40 mm (39). The mean disability score was 12.5 ± 6. RMDQ > 4 was found in 88.1% of participants. Average work lost days was 6 ± 10 days in the previous month due to LBP (Table 2).

**Table 2:** Correlations between continuous variables and RMDQ scores in patients with CLBP at the Douala General Hospital, Cameroon

Variables	RMDQ score mean ± SD	r	*p*-value
*Age in years*	50.6 ± 12.2	0.27	0.002
*Duration of CLBP in months, median (IQR)*	33.0 (69.0)	0.09	0.305
*Duration of current pain episode in months, median (IQR)*	12.0 (21.0)	0.18	0.034
*Pain intensity*	41.3 ± 24.3	0.45	< 0.0001
*Days of work loss*	6.0 ± 10.2	0.36	< 0.0001
*Units of Alcohol per week*	5.5 ± 11.7	−0.02	0.835
*BMI (kg/m* ^*2*^ *)*	29.6 ± 5.7	−0.01	0.942
*Sleep satisfaction*	65.0 ± 22.5	−0.19	0.030
*Psychological wellbeing*	59.9 ± 15.7	**−0.40**	**< 0.0001**

*SD* = standard deviation, *r* = Pearson’s correlation coefficient, *IQR* = interquartile range, *BMI* = body mass index, *RMDQ* = Roland Morris Disability Questionnaire

### Factors influencing disability

**Fig. 2:**
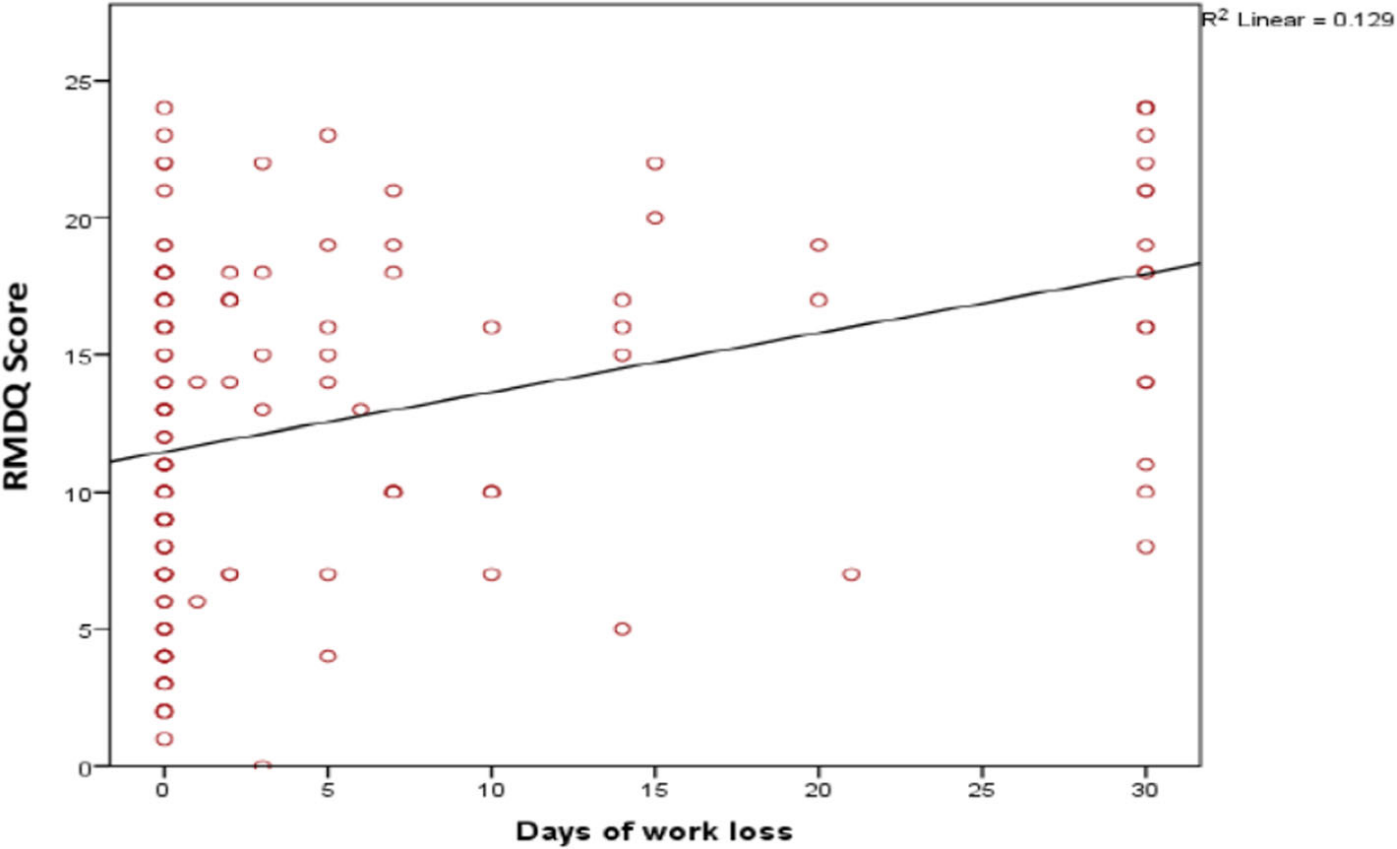
Correlation between RMDQ scores and days of work loss. Legend: Scatter plot showing correlation between disability (RMDQ scores) and days of work loss. The many small circles represent the plotted values obtained for each of the variables while the line represents the best fit for the correlation between them.

**Fig. 3:**
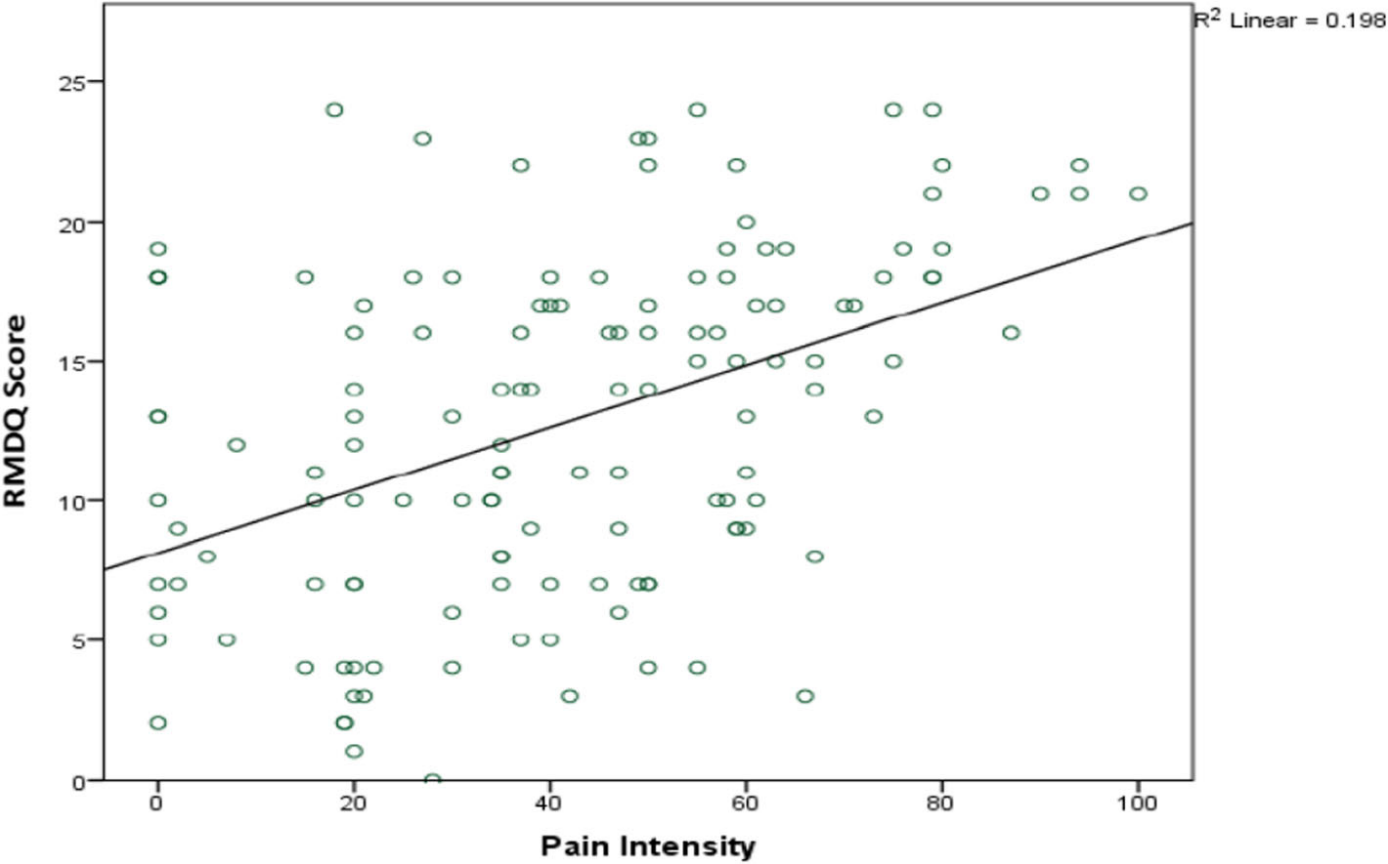
Correlation between RMDQ scores and pain intensity. Legend: Scatter plot showing relationship between disability (RMDQ scores) and pain intensity scores measured with visual analogue scale (VAS).The many small circles represent the plotted values obtained for each of the variables while the line represents the best fit for the correlation between them.

**Fig. 4:**
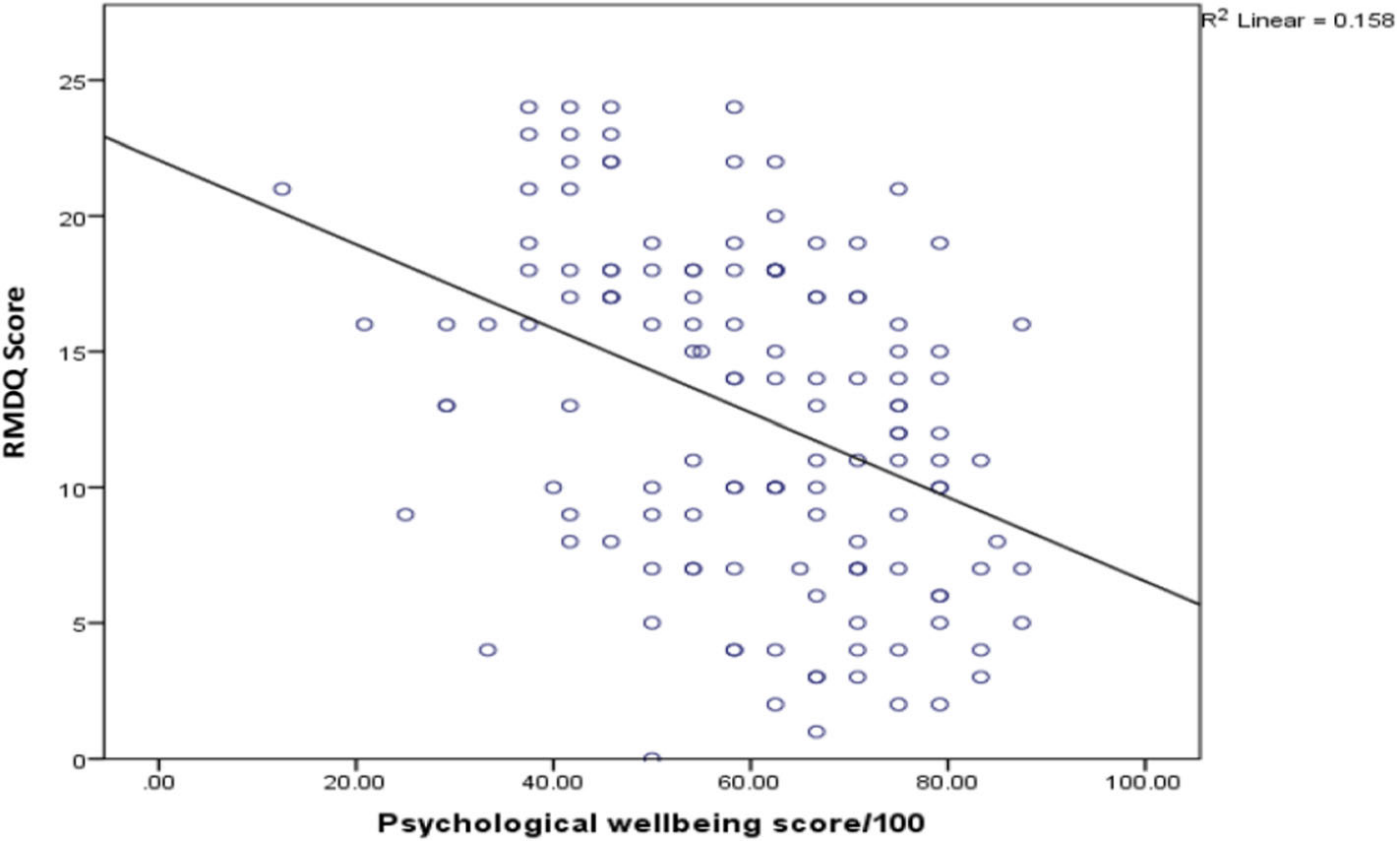
Correlation between RMDQ scores and psychological wellbeing. Legend: Scatter plot showing correlation between disability (RMDQ scores) and psychological wellbeing scores. The many small circles represent the plotted values obtained for each of the variables while the line represents the best fit for the correlation between them.

On bivariate analysis (Table 2), longer work absence (Fig. 2) and greater pain intensity (Fig. 3), moderately correlated with higher disability scores (*r* = 0.36, *p* < 0.001 and *r* = 0.45, p < 0.001 respectively). There were weak positive relationships between duration of the current pain episode (*r* = 0.18, *p* = 0.034), patients age (*r* = 0.27, *p* = 0.002) with disability. However, sleep satisfaction (*r* = − 0.19, p = 0.03) and psychological wellbeing (*r* = − 0.40, p < 0.001) negatively correlated with disability scores (Table 2 and Fig. 4).

**Fig. 5:**
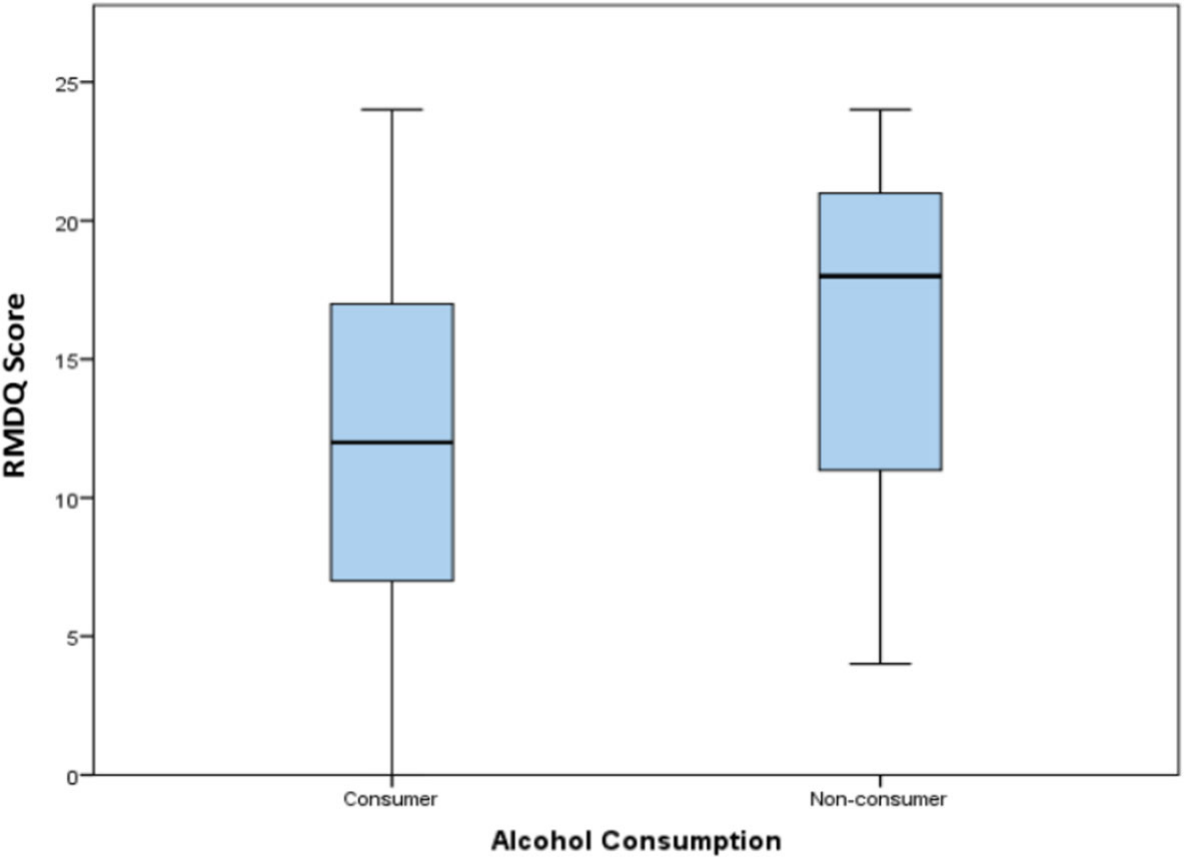
RMDQ score variation between alcohol consumers and non-consumers. Legend: Box plots showing differences in disability (RMDQ scores) by alcohol consumption status. The horizontal line in the box represents the median RMDQ score, while the lower and upper edges of the boxes represent the 25th and 75th percentiles (and interquartile range being the difference between them).The tip of the extended vertical lines on either sides of the boxes refer to the minimum and maximum RMDQ scores.

**Fig. 6:**
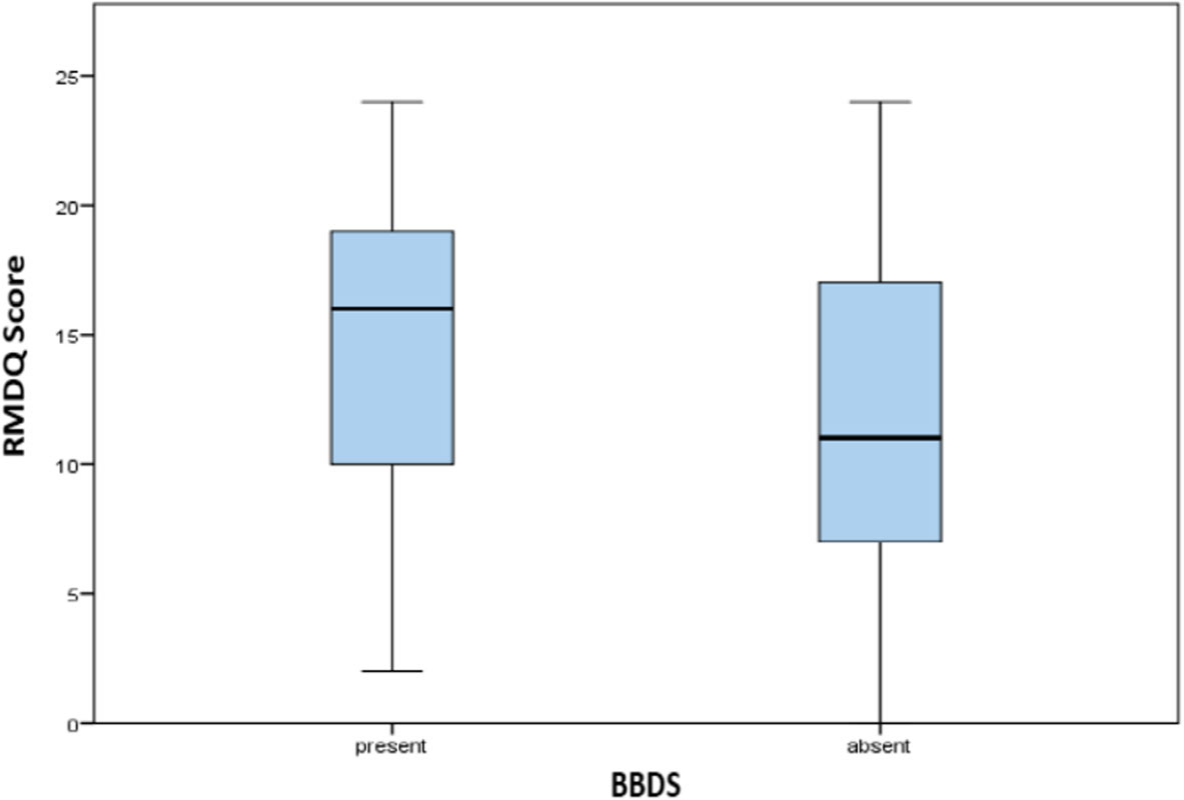
RMDQ score variation between persons with and without BBDS. Legend: Box plots showing differences in disability (RMDQ scores) in those with and without bowel/bladder dysfunction symptoms (BBDS). The horizontal line in the box represents the median RMDQ score, while the lower and upper edges of the boxes represent the 25th and 75th percentiles (and interquartile range being the difference between them).The tip of the extended vertical lines on either sides of the boxes refer to the minimum and maximum RMDQ scores.

As stated we used One-way ANOVA and employed the Tukey post hoc criterion for significance. Our findings indicated that the mean RMDQ was significantly higher in the widowed (16.9 ± 4.6) when compared to the married (12.5 ± 6.1) or single (11.5 ± 6.3); and in persons with no formal education when compared to those with tertiary education (23.0 ± 1.4 vs. 11.3 ± 5.6). Non-consumers of alcohol; persons with BBDS; and those with leg pain equally had higher mean RMDQ scores when compared with counterparts without (Table 3). Alcohol consumption (Fig. 5) and BBDS (Fig. 6) had the greatest impact on the RMDQ (*p* < 0.01).

**Table 3:** RMDQ score variations by sociodemographic and clinical characteristics of patients with CLBP at the Douala General Hospital, Cameroon

Variable	Category	RMDQ score mean ± SD	t or F statistic	*p*-value
*Gende*	Male	11.6 ± 6.4	−1.71	0.090
	Female	13.5 ± 5.9		
*Marital Status*	Married	12.5 ± 6.1^a^	**3.45**	**0.035**
	Single	11.5 ± 6.3^a^		
	Widowed	16.9 ± 4.6^b^		
*Educational level*	No formal	23.0 ± 1.4^a^	**3.67**	**0.014**
	Primary	14.3 ± 5.3		
	Secondary	12.9 ± 6.7		
	Tertiary	11.3 ± 5.6^b^		
*Employment status*	Unemployed	14.2 ± 6.5	**3.25**	**0.014**
	Employed	11.7 ± 5.8		
	Student	13.3 ± 6.0		
	Housewife	15.9 ± 6.6		
	Retired	17.4 ± 5.4		
*Employment type*	Physical labour	13.1 ± 6.8	1.18	0.313
	Non-physical	11.1 ± 5.4		
	Combination	12.8 ± 5.9		
*Income level (FCFA)*	< 50,000	13.6 ± 6.3	0.78	0.507
	50,000–100,000	12.8 ± 5.2		
	100,000 – 300,000	11.4 ± 5.9		
	> 300,000	12.4 ± 6.6		
*Alcohol*	Non-consumer	16.2 ± 5.7	**−3.31**	**0.001**
	Consumer	11.9 ± 5.9		
*Smoking*	Non-smoker	12.7 ± 5.9	0.53	0.592
	Former	12.9 ± 7.0		
	Current	16.3 ± 8.6		
*Sensory neuropathy*	Absent	11.8 ± 6.3	−1.93	0.055
	Present	13.8 ± 5.9		
*BDDS*	Absent	11.8 ± 5.9	**−2.67**	**0.008**
	Present	14.7 ± 6.2		
*Leg pain*	Absent	11.6 ± 6.4	**−2.06**	**0.041**
	Present	13.7 ± 5.8		
*Treatment*	No	10.6 ± 6.4	1.84	0.68
	Yes	13.2 ± 6.0		

^a-b^= Means in a category with unidentical superscript letters differ (*P* < 0.05), using Tukey post-hoc criterion

In the multivariable linear regression model, the factors that were independently associated disability were; pain intensity (β = 0.07, *p* = 0.002), days of work absence (β = 0.15, *p* = 0.003), psychological wellbeing (β = − 0.10, *p* = 0.004), alcohol consumption (β = − 3.55, *p* = 0.005), and bowel/bladder dysfunction (β = 2.33, *p* = 0.029), Table 4. The model explained 40.7% of the variance in disability scores.

**Table 4:** Multivariable linear regression showing factors independently associated with disability (RMDQ score) in patients with CLBP, Douala General Hospital, Cameroon

Variables	Categories	β	95% CI	*p*-value
*Marital Status*	Single	−1.31	−4.21 to 1.58	0.371
	Widowed	3.42	−0.14 to 6.98	0.059
	Married	1		
*Gender*	Female	−0.05	−2.12 to 2.02	0.963
	Male	1		
*Educational level*	Tertiary	−1.28	−9.55 to 6.99	0.759
	Secondary	−2.49	−10.85 to 5.86	0.555
	Primary	−1.98	−10.26 to 6.31	0.637
	No formal	1		
*Employment status*	Retired	−0.90	−5.87 to 4.08	0.721
	Employed	0.15	−3.13 to 3.43	0.929
	Student	−0.59	−8.08 to 6.91	0.877
	Housewife	0.62	−3.83 to 5.06	0.784
	Unemployed	1		
*Alcohol*	Consumer	−3.55	−6.01 to −1.10	0.005
	Non-consumer	1		
*Sensory neuropathy*	Present	0.67	−1.35 to 2.70	0.510
	Absent	1		
*Sphincter Dysfunction*	Present	2.33	0.25 to 4.42	0.029
	Absent	1		
*Leg pain*	Present	0.51	−1.49 to 2.52	0.613
	Absent	1		
*Age*		0.03	−0.74 to 1.30	0.585
*Pain intensity*		0.07	0.03 to 0.11	0.002
*Duration of pain episode*		−0.01	−0.03 to 0.02	0.747
*Sleep satisfaction*		−0.03	−0.07 to 0.01	0.137
*Days of work loss*		0.15	0.05 to 0.24	0.003
*Psychological wellbeing*		−0.10	−0.16 to −0.03	0.004

*aR*^*2*^ = 0.407, *β* = beta coefficient, *CI* = confidence interval

## Discussion

The aim of this study was to describe the level of disability and associated factors among individuals with CLBP in Cameroon. In this hospital-based study, we found that more than 80% of CLBP patients had significant disability. Affected individuals reported on average six days of absence from work in previous month due to their back pain thus suggesting that CLBP has a considerable impact on productivity. Factors independently associated with disability in our cohort of CLBP patients were present pain, days of work absence, psychological wellbeing, alcohol consumption and BBDS.

The mean RMDQ score in our study was 12.8 which was over 3 times the cut off for dysfunctionality proposed by Stratford and Riddle [34]. Four out of five participants had dysfunctional LBP. CLBP patients in Cameroon therefore, have high levels of functional impairment and the level of disability is consistent with findings in other settings [19, 20, 22, 27, 39]. Our results support the likely validity and applicability of the RMDQ score in the Cameroonian context.

Absence from work or restriction of routine activity is a major cause of reduced productivity and economic loss associated with LBP. We observed that CLBP accounted for over six days of absence from work in a month amongst our patients. In Taiwan a mixed cohort of acute and chronic LBP patients in ambulatory clinics reported 4.6 ± 8.4 days [39], thus in line with our results. In Uganda, the impact was higher, with up to 14 days of work loss reported in CLBP patients [11].

In a WHO multinational Study on Global Aging and Adult Health (SAGE), pain intensity was independently predictive of disability in aged adults with LBP [31]. Similarly, in Brazilian CLBP patients, pain intensity showed low to moderate correlations with the RMDQ score [20]. Mirroring these findings, pain significantly contributed to disability in our patients. However, the variability in our disability score was influenced more by alcohol consumption, sphincter dysfunction and work absence. The relationship between alcohol consumption and disability appears ambiguous. Alcohol consumption did not affect disability in the SAGE study. However, similar to our findings, a one-year cohort study exploring factors associated with disability in patients with radicular LBP found that non-consumption of alcohol was associated with greater disability [30].

Approximately one third of LBP patients have been found to have sphincter dysfunction [40, 41]. However, its contribution to disability in these patients has not yet been elaborately explored. Evidence from one study found that LBP patients with higher levels of disability were more likely to suffer urinary incontinence [40]. Our findings confirm this and demonstrate that difficulty with urinary or faecal control has a significant effect on disability in LBP.

Psychological factors are known to influence disability in LBP [19, 22, 27, 42, 43]. We observed a moderate-to-strong correlation between psychological wellbeing and disability. Furthermore, poor sleep quality has been clearly implicated in increased pain related-disability [26, 44]. In our study, this relationship was significant only in bivariate analysis.

This study had a number of limitations. First, the use of a cross-sectional study design limited the establishment of temporality or causal relationships, which would have been possible with a prospective cohort design. However, our study revealed associations that can serve as benchmark in the design of future studies. Secondly, our study may be prone to selection bias owing to the non-random sampling and hospital-based nature of the study. It is thus likely that our findings may not reflect the situation of CLBP patients at other health facilities across the county. Interpretation of our results in terms of generalizability must therefore be done with caution.

Nevertheless, we have used rigorous statistical methods to explore the burden and factors associated with disability in patients with chronic low back pain. Our study to the best of our knowledge is the first in Cameroon to investigate the disability and chronic low back pain relationship and thus may serve as framework for further research.

## Conclusion

Evidence from this study has confirmed that CLBP is associated with significant disability and work loss. Improved psychological wellbeing was associated with less disability, while longer work absence, BBDS and not consuming alcohol were associated with greater disability. Our findings provided context-specific evidence to guide priority setting in prevention and treatment strategies to reduce the burden of low back pain. Larger and robust population-based studies are warranted to fine-tune our findings.
